# Microcirculatory dysfunction in cardiogenic shock

**DOI:** 10.1186/s13613-023-01130-z

**Published:** 2023-05-06

**Authors:** Hamid Merdji, Bruno Levy, Christian Jung, Can Ince, Martin Siegemund, Ferhat Meziani

**Affiliations:** 1 Intensive Care Unit, Department of Acute Medicine, University Hospital, Basel, Switzerland; 2 Department of Clinical Research, University of Basel, Basel, Switzerland; 3 Institut Lorrain du Cœur et des Vaisseaux, Medical Intensive Care Unit Brabois, Université de Lorraine, CHRU de Nancy, INSERM U1116, Nancy, France; 4 Division of Cardiology, Pulmonology, and Vascular Medicine, Medical Faculty, University Hospital Düsseldorf, Heinrich-Heine-University, 40225 Düsseldorf, Germany; 5 Department of Intensive Care, Erasmus MC, University Medical Center, Rotterdam, The Netherlands; 6 Faculté de Médecine, Université de Strasbourg (UNISTRA), Strasbourg, France; 7 Service de Médecine Intensive-Réanimation, Hôpitaux Universitaires de Strasbourg, Nouvel Hôpital Civil, 1, Place de L’Hôpital, 67091 Strasbourg Cedex, France; 8 INSERM (French National Institute of Health and Medical Research), UMR 1260, Regenerative Nanomedicine (RNM), FMTS, Strasbourg, France

**Keywords:** Cardiogenic shock, Heart failure, Microcirculation, Macrocirculation, Perfusion parameters

## Abstract

Cardiogenic shock is usually defined as primary cardiac dysfunction with low cardiac output leading to critical organ hypoperfusion, and tissue hypoxia, resulting in high mortality rate between 40% and 50% despite recent advances. Many studies have now evidenced that cardiogenic shock not only involves systemic macrocirculation, such as blood pressure, left ventricular ejection fraction, or cardiac output, but also involves significant systemic microcirculatory abnormalities which seem strongly associated with the outcome. Although microcirculation has been widely studied in the context of septic shock showing heterogeneous alterations with clear evidence of macro and microcirculation uncoupling, there is now a growing body of literature focusing on cardiogenic shock states. Even if there is currently no consensus regarding the treatment of microcirculatory disturbances in cardiogenic shock, some treatments seem to show a benefit. Furthermore, a better understanding of the underlying pathophysiology may provide hypotheses for future studies aiming to improve cardiogenic shock prognosis.

**Graphical Abstract:**

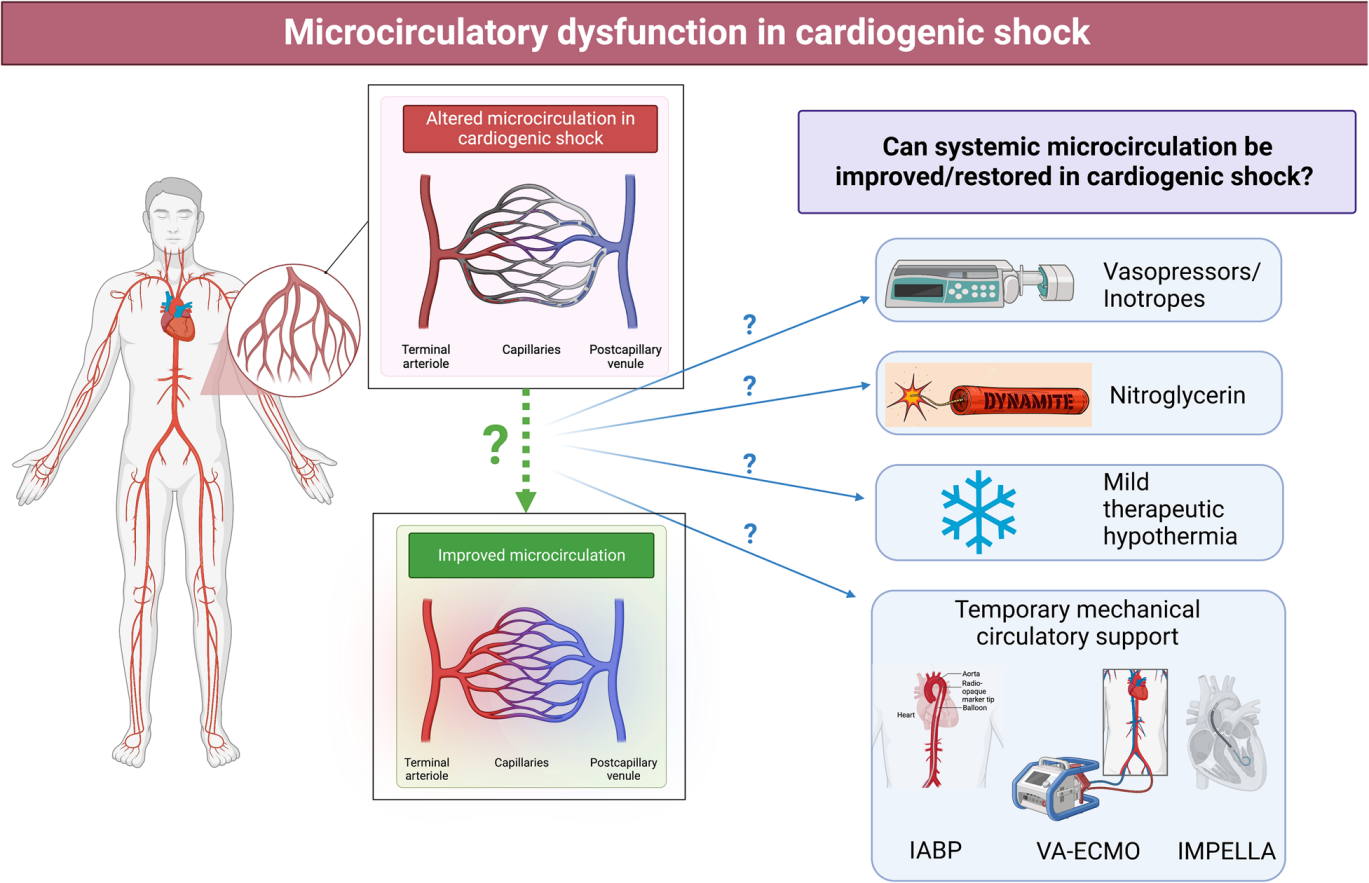

## Highlights

- Most reviews about cardiogenic shock still focus mainly on systemic macrocirculation parameters, such as blood pressure, left ventricular ejection fraction, or cardiac index to explain the pathophysiology.
- However, mortality and outcomes in cardiogenic shock are also strongly associated with microcirculation disorders that are not necessarily correlated with those of the macrocirculation.
- Although microcirculation has been widely studied in the context of septic shock, there is now a growing body of literature focusing on cardiogenic shock.

## Introduction

To date, even if there is no precise uniform definition of cardiogenic shock (CS), it is generally considered as a state of tissue and end-organ hypoperfusion caused by an ineffective cardiac output (CO) unable to deliver sufficient oxygen to organs and peripheral tissues fulfilling metabolic demands, assumed that intravascular volume is adequate [1, 2]. This inadequate end-organ perfusion associated with microcirculatory dysfunction and multiple organ failure is included in all current definitions of CS as “*signs of poor peripheral tissue perfusion*”, such as cold extremities, mottling, elevated capillary refill time (CRT), altered mental status, oliguria or elevated arterial lactate levels [3]. However, only recently have studies attempted to better characterize the microcirculatory dysfunction in CS [4].

**Fig. 1:**
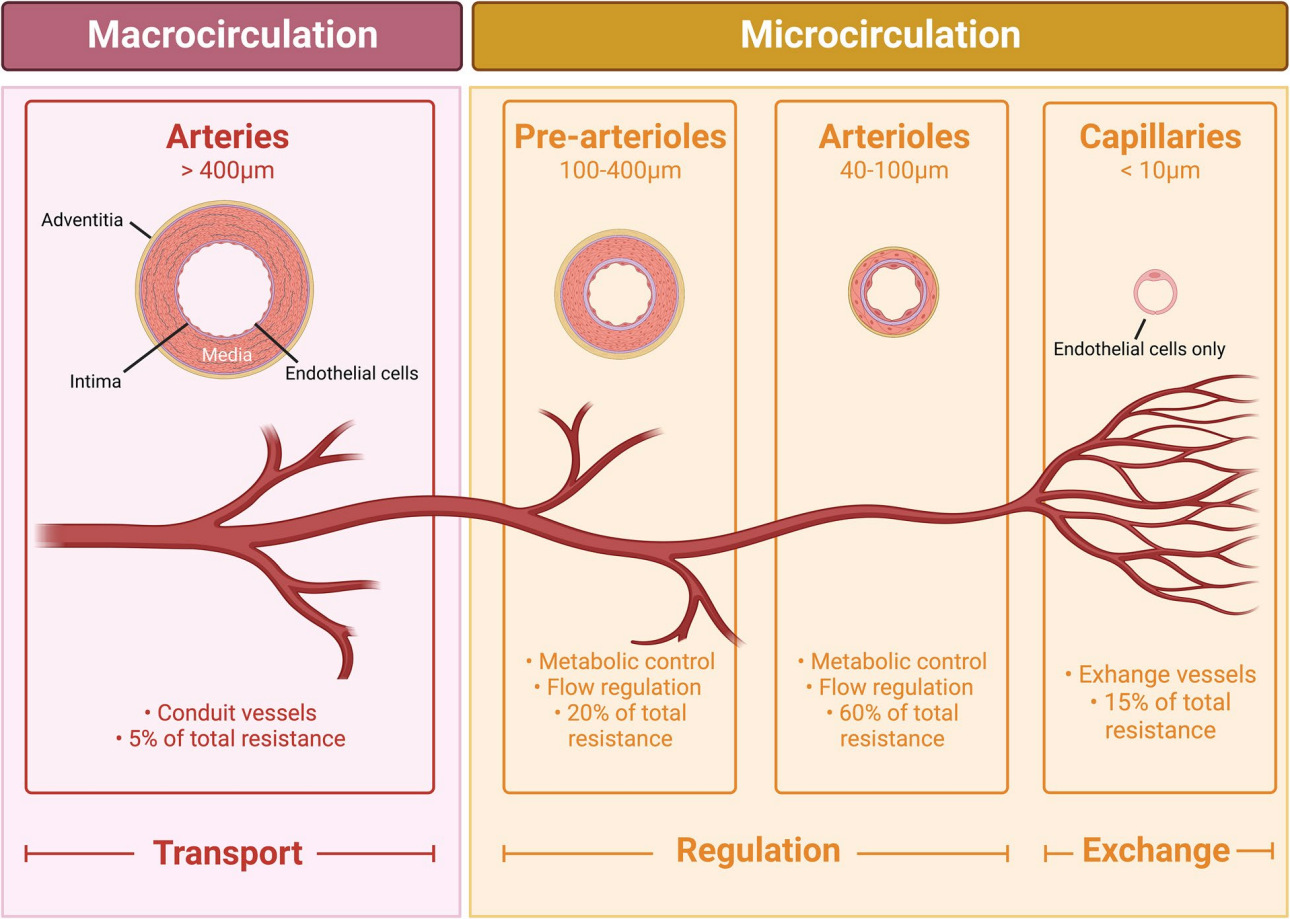
Microcirculation structure and function. The organ vasculature system has been anatomically and functionally subclassified into macro and microcirculation. Macrocirculation is constituted by conduction arteries (such as the aorta) before entering the resistance arteries (such as the mesenteric arteries) with the main purpose of transporting blood. Microcirculation is composed of pre-arterioles and arterioles regulating blood flow, leading to capillaries allowing the exchange of gases, nutrients, hormones, and other molecules

Many studies showed that CS not only involves systemic macrocirculation abnormalities, such as blood pressure (BP), left ventricular ejection fraction (LVEF), or CO [5], but also significant abnormalities of the systemic microcirculation [6, 7]. Indeed, despite progress in the management of CS, in particular by promptly restoring macro-hemodynamics, mortality remains high [8, 9]. Some studies even report that up to 45% of patients dying from CS have a normalized cardiac index (CI) (*i.e.*, > 2.2 L/min/m^2^), indicating that optimization of macrocirculatory parameters alone is not enough [10]. This may be in part explained by organ-perfusion disorders that extend beyond the macrocirculation and subsequently drive multiple organ failures. The state where main macrocirculation parameters such as BP and CI are restored, while microcirculation parameters are not, is called “loss of hemodynamic coherence”. Indeed, in CS, vascular regulation and compensatory mechanisms needed to sustain hemodynamic coherence appear to be lost in most cases, resulting in regional microcirculation remaining in shock. This so-called “loss of hemodynamic coherence” between macrohemodynamic and microhemodynamic parameters evidences that microvascular perfusion is one of the major determinants of clinical outcome in CS [11, 12]. Microcirculation is a complex system regulating the balance between tissues' oxygen consumption and delivery (Fig. 1) [13]**.** So far, microcirculatory disorders have been widely explored in the context of intensive care medicine, mostly in septic shock, showing highly heterogeneous alterations with clear evidence of arteriolar–venular shunting [14, 15] in different tissues including the lungs, the kidneys, the liver, the gastrointestinal tract and the brain [16]. Further studies are, therefore, still necessary focusing exclusively on microcirculation dysfunction in CS and its specificities [17]. Despite the paucity of clinical data on microcirculation-enhancing therapies to date, a better understanding of these dysfunctions might help improve CS management in the future. Thus, this narrative review article will focus on systemic microcirculatory dysfunction in CS and its specificities. This review will not discuss specific coronary microcirculation alteration, especially during acute myocardial infarction (AMI) which is beyond the scope of this review [18].

### Epidemiology of cardiogenic shock

Cardiogenic shock incidence has been constantly increasing for several years in United States of America and Europe, now accounting for almost 8% of admissions in ICU [19]. Although Harrison introduced CS as a specific entity in 1939 and differentiated it from other forms of shock, CS remains nowadays one of the greatest challenges in cardiology and intensive care medicine. Cardiogenic shock is the most severe manifestation of AHF, accounting for < 5% of acute heart failure (AHF) cases in the western world [20]. Compared to AHF, CS has tenfold higher in-hospital mortality, remaining > 40% despite recent advances [21, 22]. Unlike CS, patients with AHF do not exhibit prolonged hypotension with systolic blood pressure (SBP) < 90 mmHg and do not require vasopressors to raise SBP > 90 mmHg in the absence of hypovolemia (Table 1) [23]. In contrast to AHF, CS mainly shows signs of hypoperfusion, such as increased capillary refill time, mottling, cold periphery or clammy skin, confusion, oliguria, and elevated serum lactate [23]. Indeed, studies report that CS main clinical presentations are mostly wet-cold (∼65%) and dry-cold (∼30%) (“cold” meaning hypoperfusion), while AHF has signs of hypoperfusion in less than 20% of the cases usually [24, 25].

**Table 1:** Main differences between acute heart failure and cardiogenic shock

	Acute heart failure	Cardiogenic shock
Onset	• Days (*e.g.,* acute decompensated heart failure) • Hours (*e.g.,* acute pulmonary oedema)	• Hours
Blood pressure	• SBP > 90 mmHg	• Life-threatening hypotension with SBP < 90 mmHg or MAP < 60 mmHg • BP may be initially preserved by compensatory vasoconstriction
Cardiac index (CI)	• CI > 2.2 L/min/m^2^ usually	• Low CI ≤ 2.2 L/min/m^2^
Hypoperfusion and organ dysfunction	• Sometimes	• Always
Main clinical presentations	• Wet-warm (∼70%) • Wet-cold (∼20%)	• Wet-cold (∼65%) • Dry-cold (∼30%)
Need for vasopressors/inotropes to achieve and maintain a target SBP > 90 mmHg or MAP ≥ 65 mmHg	• No	• Yes
Arterial lactate	• < 2 mmol/L usually	• ≥ 2 mmol/L
pH level	• Normal pH usually	• Metabolic acidosis
Consider temporary MCS	• Rarely (*e.g.,* “protected PCI” with Impella)	• Sometimes

This main clinical presentation is based on bedside evaluation and categorization by clinical signs of congestion (‘wet’ vs. ‘dry’ if present vs. absent) and hypoperfusion (‘cold’ vs. ‘warm’ if present vs. absent)

*CI* cardiac index, *MAP* mean arterial pressure, *MCS* mechanical circulatory support, *SBP* systolic blood pressure

### From heart to microcirculation via macrocirculation: the cardio-vascular continuum

Once ejected by the left ventricle, the oxygenated blood will progressively pass through conductance arteries (such as the aorta) before entering resistance arteries (such as mesenteric arteries) and then will reach the microcirculation [13].

Microcirculation is the terminal vascular network of systemic circulation consisting of microvessels with diameters < 20 μm including arterioles, capillaries, and venules [26] (Fig. 1), Altogether, it represents the largest vascular surface area in the body. This part of the circulation is critical as it is responsible for oxygen transfer and nutrient delivery from the erythrocytes in the capillaries to the parenchymal cells to meet their metabolic demands. Microcirculation is also involved in regulating blood flow and tissue perfusion in response to hemodynamic alterations, to tailor oxygen delivery across microvascular beds with different oxygen needs. In addition, microcirculation has a central role in the immune system including hemostasis via mechanisms, such as immunothrombosis [27, 28]. Two main primary factors ensure oxygen transport by erythrocyte flow in the microcirculation to the tissues. First, capillary blood flow is a complex product of arteriolar tone, driving pressure, and hemorheology allowing convection of oxygen-carrying erythrocytes (convective capacity). The second is capillary patency, reflected by functional capillary density. This functional capillary density represents the number of normally perfused capillaries in a given tissue area (diffusive capacity).

The performance of organs and tissues is, therefore, critically dependent on a functional microcapillary network that maintains delivery of oxygen, exchanges heat, and nutrients, and removes carbon dioxide and waste products [29]. Of note, a decline in capillary density might be one of the major causes of aging and age-related diseases [30].

Under physiological conditions, blood arrives microcirculation through pre-arterioles (100–400 µm in diameter) before reaching arterioles (10–50 µm in diameter), which are both surrounded by a thick, continuous layer of smooth muscle. Contraction of the smooth muscle reduces the lumen of these microvessels and, therefore, increases the resistance to blood flow throughout the entire vascular bed, making the arteriole the major resistance component in the circulation and the main driver of the total peripheral resistance. Smooth muscle tone in the arterioles also regulates the amount of pressure transmitted from the arteries to the veins; thus, capillary pressure decreases when the arterioles contract and increases when the arterioles dilate.

Further to the arterioles, the blood then enters a narrower vessel, the metarteriole (10–20 µm), which is the terminal end of the arteriole surrounded by a discontinuous smooth muscle layer. From the metarteriole, capillaries (5–10 µm in diameter and length of 5 mm), a single layer of epithelium, and a basement membrane arise and branch off. Capillary density, which is an important determinant of the total surface area available for blood–tissue exchange, varies considerably from one organ to another depending on the metabolic requirement. In human tissue, the average capillary density is around 600 per mm^3^, but it is higher in brain, lung, kidneys, liver, and myocardium (around 2500–3000 per mm^3^), reduced in phasic skeletal muscle (around 300–400 per mm^3^) and even lower in the bones, fat, connective tissues and in tonic skeletal muscle (less than 100 per mm^3^) [31].

At the junction between the metarteriole and some capillaries, a precapillary sphincter consisting of a single band of smooth muscle may be present that allows regulation of the percentage of capillaries open to erythrocyte perfusion. However, even if such precapillary sphincters have been known for decades, their existence, except within the mesentery [32] and the brain [33], remains controversial [34]. In some tissues, such as the heart, all capillaries are usually open to perfusion, whereas, in some other tissues, such as skeletal muscle and intestine, only 20–30% of capillaries are open.

In case of need, relaxation of the precapillary sphincter in the latter tissues allows for the recruitment of more open capillaries and, therefore, an increased transcapillary exchange. Finally, capillaries merge into a venule (~ 10–50 µm), which has a discontinuous, thin layer of smooth muscle draining into small veins. Changes in venous smooth muscle tone can significantly affect capillary exchange as constriction of the venules leads to an increase in capillary pressure, whereas dilation of the venules exerts the opposite effect.

One other important characteristic of microcirculation is the decrease of hematocrit in the capillaries, known as the Fåhræus effect [35]. Indeed, concentration of fast-flowing red blood cells in the center of the lumen, and of slower-flowing plasma along the wall of the vessel, in combination with plasma skimming at bifurcations [36] leads to a reduced red blood cell transit time and a decreased hematocrit in branching capillary networks. Recent data found that the Fåhræus effect may increase in shock states (reducing hematocrit even more) and thus could contribute to further decreased tissue oxygenation in low perfusion areas [37].

All the vessels of macro- and microcirculation are almost entirely lined by endothelial cells (EC) which are organ-specific. These EC help maintain organ homeostasis by regulating various functions including the trafficking of fluid, solutes, hormones, and macromolecules [38]. Frydland et al. reported that AMICS patients had a higher concentration of soluble thrombomodulin than AMI patients without CS, reflecting endothelial damage [39].

Located between the bloodstream and the endothelium, the endothelial glycocalyx is an important determinant of vascular homeostasis, composed of macromolecules such as proteoglycans and sialoprotein and also organ- and vascular bed-specific [40]. The glycocalyx is a 0.2–0.5 μm-thick gel-like layer lining the luminal membrane of the endothelium, which is considered to compromise approximately 20% of the intravascular volume. It is a multi-component layer composed of proteoglycans (including syndecan-1) and glycoproteins, anchored to the endothelium by glycosaminoglycans. Although its role in vascular permeability has recently been debated [41], the glycocalyx mediates several key physiological processes, such as vascular barrier function, hemostasis, autoregulation, leukocyte, and platelet adhesion, and also transmission of shear stress to the underlying endothelium [42]. Jung et al. showed that high syndecan-1 levels, reflecting glycocalyx shedding, were predictive of short-term mortality in early AMICS [43].

Finally, a crucial but under-investigated parameter is the interaction between microcirculation and the lymphatic system. Lymphatic vessels are present in almost all tissues (except bone marrow, cartilage, and cornea [44]) and their primary function is to drain interstitial fluid and macromolecules to the venous circulation at a total volume of almost 8 L/day [45]. In congestive heart failure, such as CS, lymphatic contractile dysfunction has been suggested to play an important role to generation of interstitial edema, causing impairment of blood flow, increasing diffusion distance, and cellular hypoxia [46]. However, there are currently no specific drug treatments in clinical use available to reduce lymphatic pump dysfunction [47].

### Microvascular flow regulation

Vasoregulation within the microcirculation itself varies according to the anatomic topography. Indeed, some of the vessels of the microcirculation are supported by vascular smooth muscle (VSM) and others are not. The VSM tone is partly modulated by local concentrations of vasoactive metabolites and mediators, autonomic influences (sympathetic stimulation causes vasoconstriction), and hemodynamic factors, but also by conducted responses from downstream vessels [48]. Increases in transmural pressure also activate mechanosensitive ion channels in VSM leading to vasoconstriction, known as the myogenic response [49].

In addition, the whole microcirculation (even not surrounded by VSM) is also affected by hemodynamic factors in responses to shear stress and circumferential wall stress generated by transmural pressure. EC sensed increases in shear stress, which leads to vasodilation due to the release of mediators including nitric oxide (NO), prostaglandins, and EDHF (endothelium-derived hyperpolarizing factor). Under hypoxic conditions, EC can also release adenosine, a potent vasodilator [48].

Thus, because the capillaries are deprived of musculature and innervation, the flow in each capillary bed is mostly driven by the hemodynamic pressures differences between the arteriolar pressure/precapillary sphincter and the postcapillary venules, also named the microcirculatory driving pressure. This condition is frequently beneficial, because a single capillary bed can be supplied by multiple arterioles, which may allow blood flow to increase by 200–500% without any significant change in overall arteriolar pressure [50]. For instance, the density of perfused capillaries may increase from 1000 to 4000/mm^2^ in the myocardium during maximal workload [51]. However, because the main pressure drastically decreases in the arterioles (resistance vessels), microcirculation at the capillary level is considered a very low-pressure compartment. Therefore, mean capillary pressure appears to be more influenced by the downstream venous pressure than the upstream arterial pressure. In this perspective, central venous pressure appears to be one of the main determinants of capillary blood flow. This is of particular concern in CS, where the central venous pressure is often very elevated [52].

Finally, oxygen pressures can be lower in the microcirculation than that of the venous oxygen levels due to shunting of the oxygen transport of the microcirculation from the arterial to the venous compartment which is why monitoring the microcirculation directly is important in identifying its dysfunction [14].

### Assessing the microcirculation

Nowadays, both direct and indirect methods are available to assess microcirculation. Each of these methods possesses advantages and disadvantages.

Direct observation of the microcirculation can be done at the bedside, using hand-held vital microscopy, such as Sidestream Dark-Field (SDF), and Incident Dark-Field (IDF) imaging techniques to assess the sublingual microcirculation [53].

Analyses of the sublingual microcirculation images allow assessments of the convective and diffusive components of the microcirculation [6]. The convective component of these functional parameters of the microcirculation can be described either semi-quantitatively, by the microcirculatory flow index (MFI), or quantitatively, by the use of space–time diagrams. The diffusive component can be described either by a combination of the De Backer score and proportion of perfused vessels (PPV), the total vessel density (TVD) if all vessels are perfused, or the perfused vessel density (PVD). The heterogeneity index reflects heterogeneities in microcirculatory flow caused by endothelial and/or erythrocyte alterations [53]. Other devices also exist using near-infrared spectroscopy (NIRS) or assessment of skin blood flow using skin laser Doppler imaging [54]. However, these technologies have many limitations [55], among them, limited availability of these different devices, lack of a clearly defined target value, and limited representativeness of microcirculatory impairment in other tissues [55].

Indirect assessment of the microcirculation can be roughly done by arterial lactate level and its variations; however, due to its well-known limitations [56], it has a poor correlation with microcirculatory disorders at the organ level [7]. Urine output has also been considered a traditional marker of tissue perfusion [57] partially reflecting microcirculation; however, it may take time to assess, and because diuretics are often used in congestion and because type 1 acute cardiorenal syndrome are frequent in CS, it may be difficult to integrate. Interestingly, surrogate indirect microcirculation assessment can also be done at the bedside using traditional markers of peripheral tissue perfusion signs, such as capillary refill time (CRT), mottling, and $\Delta$ PCO_2_ [58]. These perfusion signs are strongly linked with microcirculatory blood flow alteration in cardiogenic shock [59]. CRT measures the time required to recolor the tip of a finger. Mottling is defined as patchy skin discoloration that usually starts around the knees. Central venous–arterial carbon dioxide difference ($\Delta$ PCO_2_), also named Pv-aCO_2_ or PCO_2_ gap, is the difference between partial pressure of CO_2_ in venous blood and arterial blood [60, 61]. Although controversial, Ospina-Tascon [60], have well-highlighted the good correlation between the PCO_2_ gap and microvascular blood flow during the early phases of septic shock. However, this marker has some limitations and may vary depending on specific conditions (HbO_2_ saturation [i.e., the Haldane effect], arterial pH, temperature, and hematocrit) [61, 62].

Most of these perfusion parameters, such as CRT, have been validated with good reproducibility and excellent interrater concordance [63]. Moreover, they are simple noninvasive, priceless tools allowing a real-time assessment of microcirculation at bedside; although, in contrast to analysis of hand-held vital microscopy images, they do not give insight into underlying mechanisms associated with microcirculatory alterations [53]. Of note, comparing different peripheral tissue perfusion parameters in CS, the less relevant seemed to be the central-to-peripheral temperature difference, which is the difference between central temperature and peripheral temperature [59], although it was the first variable related to the use of the peripheral perfusion as an indicator of circulatory shock, introduced by Weil in the sixties [64].

### Microcirculation alteration during cardiogenic shock (Fig. 2)

**Fig. 2:**
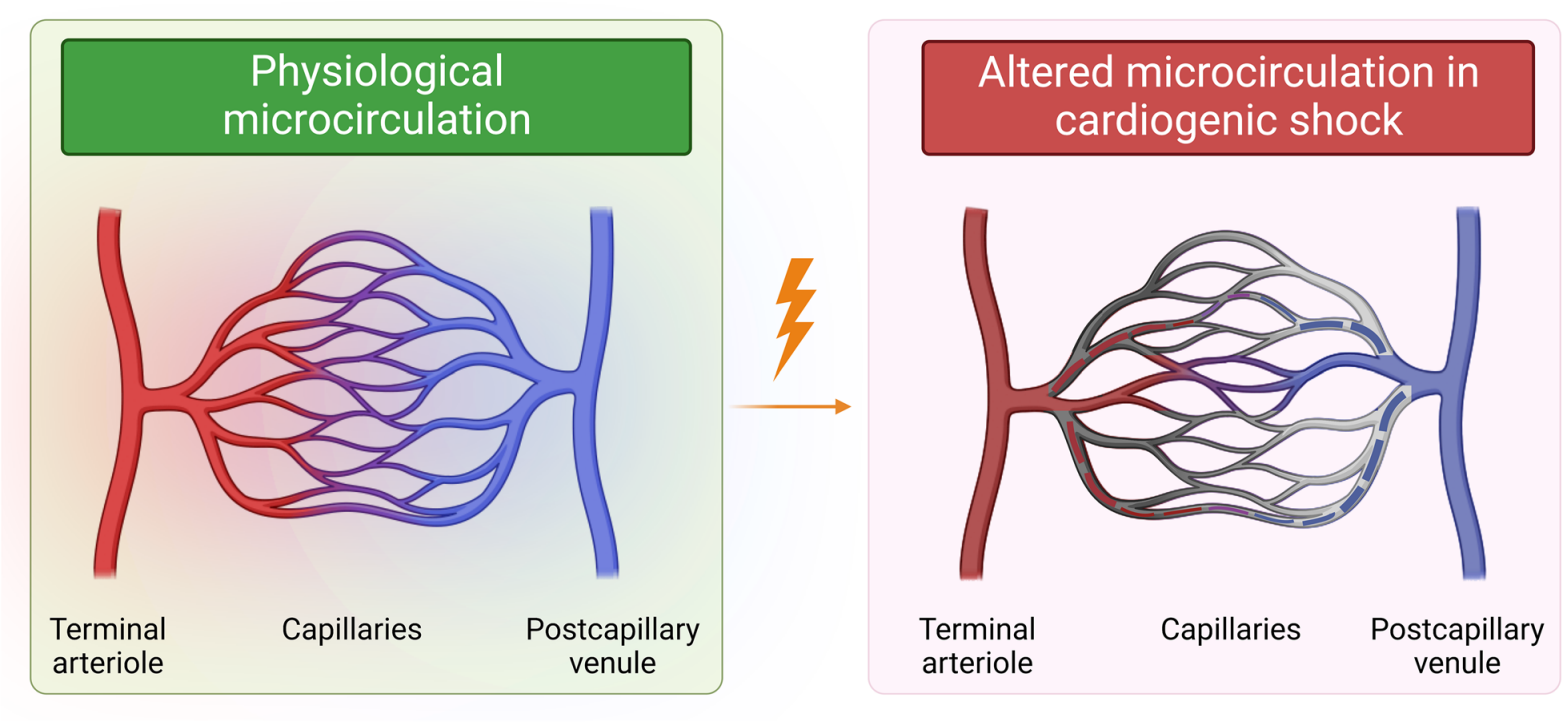
Microcirculation alteration during cardiogenic shock. Alterations of microcirculation can be characterized by multiple different types of impairments, such as no capillary perfusion, low perfusion, heterogeneous perfusion, stasis, or shunting area. Besides, it can also be a result of hemodilution of microcirculatory blood by plasma skimming resulting in the loss of erythrocyte-filled capillaries which decreases tissue oxygen delivery. Or it can be secondary to edema caused by capillary leak syndrome (seen in critically ill patients) which results in increased diffusive distance and reduced ability of the oxygen to reach the tissue cells

In 1922, Freedlander et al*.* were the first to describe altered microcirculation in patients with cardiac failure using nailfold videomicroscopy [65]; however, this site is particularly sensitive to small changes in external temperature. Even though this work was done about 100 years ago, it was not until the beginning of the twenty-first century that physicians became seriously interested in microcirculation in CS. Although the number of studies about this issue remains very limited in indexed databases, such as PubMed to date. In 2000, using venous air plethysmography, Kirschenbaum et al., measured forearm blood flow in patients with CS before and after arterial occlusion. The authors reported an attenuated vascular response to reactive hyperemia, which indicates attenuation of the microvascular response to hypoxia [66]. Indeed, a normal physiological response to reactive hyperemia is usually characterized by an increase in blood flow either from capillary recruitment and/or increased velocity of blood flow through previously opened capillaries [67]. Using modern sublingual videomicroscopy, De Backer et *al.* showed a high prevalence of microvascular blood flow alterations in patients with severe heart failure and CS [6]. These alterations included a nearly 50% decreased density of small perfused vessels with numerous non-perfused or intermittently perfused small vessels in CS compared to control patients. A marked heterogeneity was also observed between the different areas. These alterations were also more severe in patients who did not survive. Similarly, Jung et al*.*, reported reduced microvascular perfusion in patients with CS, associated with an increased arterial lactate level [7]. In a prospective cohort study of patients with AMICS, low perfused capillary density at admission was strongly and independently associated with 30-day mortality, with a greater predictive value than the baseline SOFA score [12]. Moreover, an increase in perfused capillary density after 24 h was significantly associated with a better outcome. Interestingly, decreased capillary blood flow was not correlated with standard macrocirculatory parameters, such as heart rate, blood pressure, CI, and cardiac power index (CPI) at admission. However, it was correlated with pulmonary artery occlusion pressure (PAOP).

Recently, a sub-study of the CULPRIT–SHOCK trial assessed the sublingual capillary network using videomicroscopy post-percutaneous coronary intervention [68]. The study shows that microcirculatory perfusion parameters have better prognostic value than macrocirculatory parameters to predict the combined clinical endpoint of 30-day all-cause death and renal replacement therapy in patients with AMICS. The authors demonstrated that post-percutaneous coronary intervention (PCI) normotensive CS patients with impaired microvascular perfusion have a significantly higher risk of mortality or renal replacement therapy than normotensive CS patients with normal microvascular perfusion. This loss of hemodynamic coherence between macrocirculation and microcirculatory perfusion parameters supports that microvascular perfusion may be a significant determinant for clinical outcome after AMICS, even in normotensive CS patients when macrohemodynamic conditions are restored.

These microcirculatory dysfunctions were also seen using videomicroscopy in patients with CS under veno-arterial extracorporeal membrane oxygenation (VA-ECMO) support [69–71]. In a retrospective study based on an indirect perfusion parameter strongly linked with microcirculation, a PCO_2_ gap > 6 mmHg 6 h after VA-ECMO initiation was associated with early death (under VA-ECMO or less than 72 h after VA-ECMO weaning) [72]. This increase in the PCO_2_ gap cannot be explained by inadequate hemodynamic support, as the VA ECMO flow rates and mean arterial pressure (MAP) were similar in both groups, and only a weak correlation was found between VA-ECMO flow rate and the PCO_2_ gap.

Based on easier-to-assess microcirculation parameters, the FRENSHOCK prospective study reported that mottling at admission for CS was significantly associated with 30-day mortality [73]. In another prospective observational study of CS patients, a CRT > 3 sec at the fingertip at admission in ICU was associated with an increase 90-mortality or need for VA-ECMO support. Furthermore, the combination of CardShock score with CRT > 3 sec resulted in a greater performance to predict 90-day mortality or VA-ECMO support than CardShock score alone, improving the AUC to 0.93. CRT was also well-correlated with arterial lactate and mottling but performed even better than mottling in predicting poor outcomes. Finally, in the same study, a high PCO_2_ gap seemed to be associated with poor outcomes in cardiogenic shock [59].

All of these microvascular alterations may be explained by a decrease microcirculatory driving pressure (defined as the difference between post-arteriolar and venular pressure) due to an increase in central venous pressure during CS, which may act as an outflow obstruction of organ perfusion [74]. They may also be explained by an increase in various inflammatory mediators released during CS leading to impaired leukocyte [75] and erythrocyte [66] deformability with increased attachment to vessel walls reducing microvascular flow but also leading to transudation of fluids into the perivascular region favoring interstitial edema which increases extravascular tissue pressure and changes the viscosity within the vessel lumen.

Low systemic vascular resistance or vasopressors, used to counteract this vasoplegia [76], may also be responsible for the decrease in microvascular perfusion. Vasopressor may also decrease CO by increasing the afterload of an already failing left ventricle. However, De backer et al*.* did not observe any relationship between the doses of vasoactive agents and microvascular alterations [6], whereas Jung et *al.* found an inverse correlation [77]. Finally, activation of the coagulation cascade and formation of microthrombi obstructing the microcirculation have been suggested but are unlikely because microvascular alterations were also seen in patients treated with multiple anti-aggregation therapies and anticoagulant drugs for AMICS [7, 12].

As a concrete illustration, impairment of the microcirculation within the lungs may cause the activation of arteriovenous shunts, ultimately leading to the development of atelectasis and hypoxemia [78, 79]. While altered microcirculation in the liver may result in functional disturbances, such as impaired synthesis of coagulation factors [4]. Consequently, acute hepatic dysfunction, also known as “shock liver,” results in reduced synthesis of protein C and antithrombin, which predisposes the individual to microvascular thrombosis [80]. In the gastrointestinal tract, microcirculatory disorders during experimental autoimmune myocarditis have been found to play a significant role in the deterioration of its enterocyte barrier function in mice [81]. This intestinal barrier alteration may potentially allow the translocation of bacteria or endotoxins into the bloodstream, which may contribute to vasoplegia, aggravating the initial CS state [82].

**Fig. 3:**
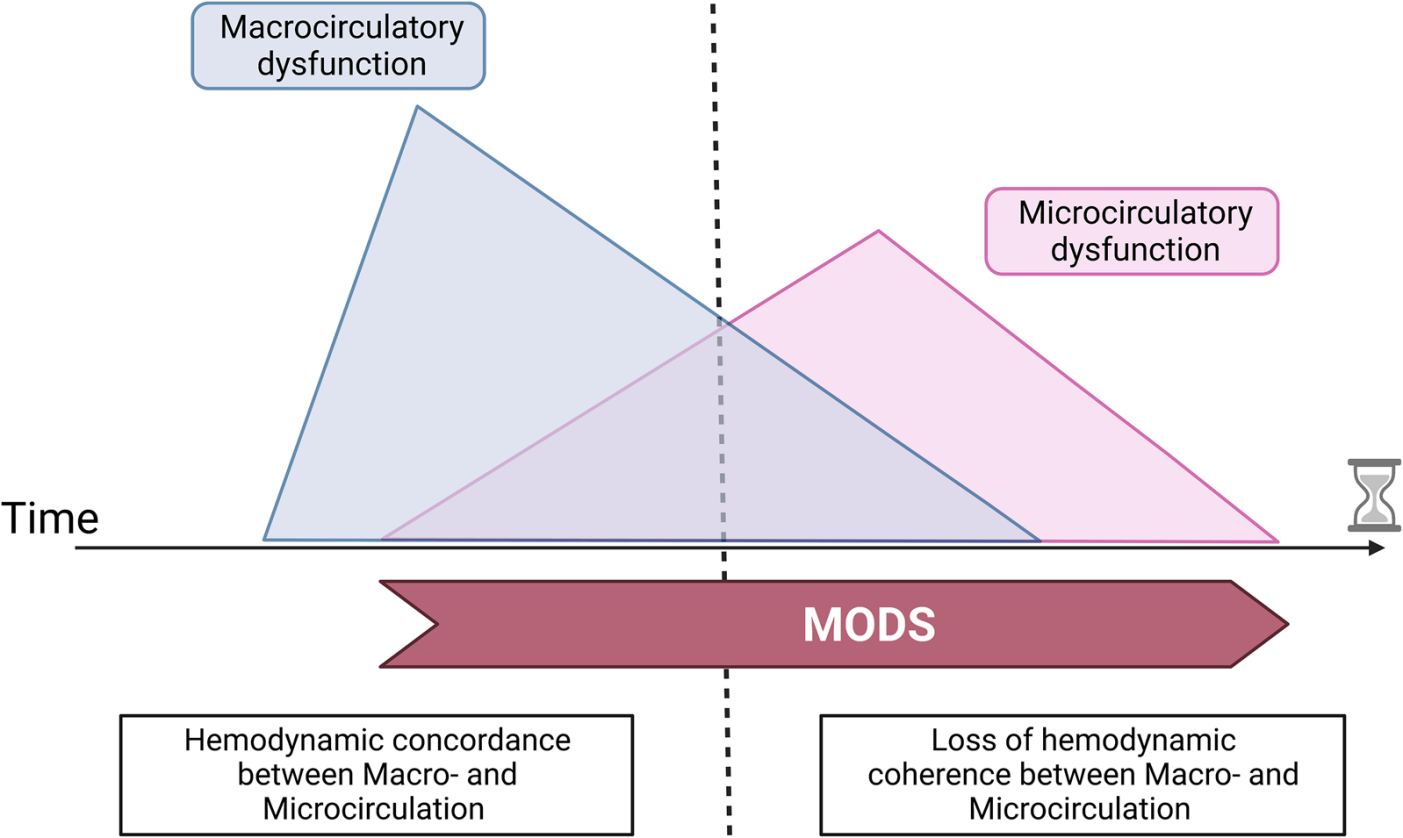
Schematic time course of macro- and microcirculatory dysfunction in cardiogenic shock (adapted from Chioncel et al., 2020). While macrocirculatory dysfunction seems to predominate initially during CS, the microcirculation becomes progressively dysfunctional in a second phase. This can ultimately lead to a loss of hemodynamic coherence. *MODS* multiple organ dysfunction syndrome

However, using sublingual SDF imaging in an experimental preclinical porcine model of CS, Stenberg et al., showed that microcirculation might be initially preserved in the first hours of CS despite severe alteration of macrocirculation parameters [83] (Fig. 3, adapted from Chioncel et al., 2020 [84]). Interestingly, in a preclinical murine model of CS, while sublingual microcirculation was rapidly altered during the initial phase of CS, the cerebral cortical microcirculatory flow remained fully preserved, at least during the first 4 h of CS [85]. These preclinical results suggest that time (potentially required to induce systemic inflammatory response syndrome) and probably ischemia–reperfusion injury may play a role.

### Can systemic microcirculation be improved in cardiogenic shock? (see Table 2)

**Table 2:** Impact on macro- and microcirculation of drug and mechanical circulatory support devices used in cardiogenic shock

Drugs/MCS devices	Mechanism of action	Study protocol	Effects on macrocirculation described in the study	Effects on microcirculation described in the study	Study [Ref]
Dobutamine	β_1_ adrenergic receptor agonist	Dobutamine was given when CI was < 2.2 L/min/m^2^ or SvO_2_ was < 65%	Increase HR, CI and SvO_2_ Slight reduction of PAOP	No effect on microcirculation	Den Uil et al., PMID: 25084171
Levosimendan	Myofilament calcium sensitizer			No study in cardiogenic shock	(An abstract, published in German in 2009 in Clin Res Cardiol seems to show improvement of microcirculation)
Milrinone	Phosphodiesterase-3 inhibitors increasing intracellular calcium by inhibiting the degradation of cAMP			No study in cardiogenic shock	
Enoximone	Phosphodiesterase-3 inhibitors increasing intracellular calcium by inhibiting the degradation of cAMP	Enoximone was given when CI was < 2.2 L/min/m^2^ or SvO_2_ was < 65%	Decrease CVP and PAOP	Increase PCD	Den Uil et al., PMID: 25084171
Norepinephrine	α_1_ and β_1_ adrenergic receptor agonist	Norepinephrine was given to patients when MAP was < 60 mmHg, independent of CI or SvO_2_, to reach a target MAP ≥ 70 mmHg	Increase MAP	Slight non-significant reduction of PCD	Den Uil et al., PMID: 25084171
		Norepinephrine was given to increase MAP from 65–70 to 80–85 mmHg	Increase MAP	Increase delta StO_2_ and StO_2_ recovery slope (NIRS)	Perez et al., PMID: 24509521
Epinephrine	Stimulates both α_1_ and β_1_ adrenergic receptors			No study in cardiogenic shock	
Nitroglycerin	Organic nitrate providing an exogenous source of NO	Infusion was started at 8 µg/min and then doubled every 30 min up to 133 µg/min	Increase CI Decrease MAP, CVP and PAOP	Increase in PCD	Den Uil et al., PMID: 19639300
IABP	Intra-aortic balloon inflating during diastole to increase coronary perfusion and deflating during systole to decrease afterload	IABP was inserted in AMICS	Effect on microcirculation not described in this study	No effect on microcirculation	Jung et al., PMID: 25720332
		IABP was withdrawn in recovering CS patients	Withdrawal of IABP led to a decrease in MAP and an increase in diastolic arterial pressure	Withdrawal of IABP led to an increased PVD	Munsterman et al., PMID: 20738876
		Intentional stop of IABP support in CS	Effect on microcirculation not described in this study	IABP stop led to a decrease MFI	Jung et al., PMID: 19367424
VA-ECMO	Percutaneous cardiopulmonary bypass providing full hemodynamic support and increasing afterload	VA-ECMO implantation in refractory CS	Reduce HR and LVEF	Increase PPV, MFI and perfused SVD	Chommeloux et al., PMID: 31634235
		Under VA-ECMO: increasing dobutamine above 5 μg/kg/min or VA-ECMO flow	While increasing dobutamine: increase HR and AoVTI While increasing VA-ECMO-flow: increase HR	No effect on microcirculation while increasing dobutamine or VA-ECMO-flow	Chommeloux et al., PMID: 35700546
		Under VA-ECMO inserted within 48 h: increasing VA-ECMO pump flow or decreasing VA-ECMO pump flow	No change in MAP while increasing VA-ECMO pump flow	Both contradictory and non-contradictory responses of sublingual microcirculation Probability of increasing PVD after increasing VA-ECMO pump flow were higher in the events with a PVD < 15 mm/mm^2^ at baseline	Wei et al., PMID: 33898485
		Under VA-ECMO in patient with MAP < 60 mmHg: inotropic and vasopressor agents (dopamine, dobutamine, norepinephrine or epinephrine) were administered to target and maintain a MAP at 60–90 mmHg	Increase HR and MAP	No differences were observed in Thenarmuscle StO_2_ and cerebral rSO_2_ Thenar muscle StO_2_ desaturation slope and resaturation slopes during the vessel obstruction test were also unchanged	Du et al., PMID: 27983541
Impella	Temporary percutaneous LVAD with a nonpulsatile axial flow pump that propels blood from the left ventricle into the ascending aorta through the catheter	Impella LP2.5 was inserted after PCI for a first anterior STEMI (No CS in this study but acute heart failure)	Increase LVEF	Increase PVD and MFI	Lam et al., PMID: 19280085

*AMI* acute myocardial infarction, *AMICS* acute myocardial infarction complicated by cardiogenic shock, *AoVTI* aortic velocity–time integral, *CI* cardiac index, *CS* cardiogenic shock, *CVP* central venous pressure, *IABP* intra-aortic balloon pump, *LVAD* left ventricular assist device, *LVEF* left ventricular ejection fraction, *MCS* mechanical circulatory support, *MFI* microvascular flow index, *PAOP* pulmonary artery occlusion pressure, *PCD* perfused capillary density, *PCI* percutaneous coronary intervention, *PPV* proportion of perfused vessel, *PVD* perfused vessel density, *SVD* small-vessel density, *STEMI* ST-element elevation myocardial infarction, *VA-ECMO* veno-arterial extracorporeal membrane oxygenation

In the study of De Backer et *al.,* the microvascular blood flow alterations in patients with severe heart failure and CS could be totally reversed with the topical application of acetylcholine (using a piece of gauze soaked with acetylcholine at a concentration of 10–2 M during 1 min) suggesting that the endothelium was still able to respond to vasodilators and that therapeutic interventions aiming at opening the microcirculation may be considered [6].

Nitroglycerin, an organic nitrate, such as isosorbide dinitrate, acts by providing an exogenous source of NO which binds to soluble guanylate cyclase, producing cyclic guanosine monophosphate (GMP) leading to vascular smooth muscle relaxation [86]. Den Uil et al*.* showed that intravenous low-dose nitroglycerin in CS was associated with an increase in sublingual perfused capillary density but also with a reduction in cardiac filling pressures (both central venous pressure and PAOP) [87]. In the present case, it is likely that nitroglycerin improved microcirculation through both macro and microcirculatory effects. However, because vasodilators induce hypotension, guidelines contraindicate their use in cases of shock with a systolic BP < 110 mmHg [23]. Another limitation is nitrate tolerance which may develop within 24 h, but this reduced effectiveness may be overcome by increasing the dosage. However, no prospective study to date has assessed vasodilators, such as nitroglycerin, in association with vasopressors, such as norepinephrine in CS. This combination which may seem counterintuitive, using a prostacyclin analog (an endothelium-derived relaxing factor), is currently being evaluated in septic shock [88]. It is noteworthy that most data show no deleterious effect of norepinephrine on microcirculation [89], which could be explained partly because capillaries consist of a single layer of epithelium and a basement membrane not surrounded by smooth muscle.

In a prospective comparative study in AHF, Teboul et *al.* showed that the PCO_2_ gap was found to decrease while increasing the dose of dobutamine from 0 to 10 μg/kg/min (p < 0.05) and then to increase slightly, but not significantly, when the dose was increased above [90].

In a sub-study of the IABP–SHOCK II trial which is the first randomized study directly investigating the microcirculation in patients with CS, Jung et *al.* assessed perfused capillary densities (< 20 µm), perfused vessel densities (< 100 µm), total capillary densities and total vessel densities using a SDF intravital microscope [77]. Although the intra-aortic balloon pump (IABP) increases MAP and CO (∼0.5 L/min), it does not improve clinical outcomes in patients with AMICS or their microcirculation. Indeed, results revealed no difference regarding the aforementioned microcirculation parameters between patients treated with or without an IABP. Munsterman et *al.* even found that IABP worsens microcirculation in patients having suffered CS, showing an increase in PVD of small vessels after withdrawal of IABP [91].

Recently, in the randomized SHOCK–COOL Trial, mild therapeutic hypothermia (24 h at 33 °C) in patients after primary percutaneous coronary intervention for AMICS did not show any substantial benefit on macro (CPI in the first instance) and microcirculation (assess using sublingual videomicroscopy) and also no clinical benefit in survival [92]. Suggesting no benefit of mild hypothermia in CS.

To date, there is very limited data showing a drug benefit, whether inotropic or vasopressor agents, on microcirculation in CS [89]. In a small study, Enoximone tested in ten CS shows a microcirculation improvement in CS [93]. Moreover, increasing MAP from 65–70 to 80–85 mmHg with norepinephrine in AMICS was associated with an improved microcirculation as assessed by thenar NIRS measurements [94]. However, most of these patients were post-cardiac arrest CS generally presenting with a shock state different from standard CS [95, 96].

In a study assessing microcirculation in refractory CS patients supported by VA-ECMO, almost all microcirculation parameters, except small vessel density, improved 12 h after VA-ECMO initiation [97]. Interestingly, in this study, the inability to rapidly normalize microcirculation parameters during the first 24 h of VA-ECMO support, despite normal macrocirculation parameters, was associated with mortality. Moreover, microcirculatory flow response as a result of 50% pump flow decrease from the baseline visualized by hand-held vital microscopy occurring during VA-ECMO reliably predicted success of weaning [69]. These results were confirmed in a study by Wei et al., however, in addition they also identified that some patients paradoxically showed a reduction in microcirculatory flow after an increase in VA-ECMO pump flow [70]. Similarly, successful improvement of perfused small vessel density within the first 24 h of VA-ECMO initiation was able to accurately predict in-ICU mortality [71].

Using NIRS, microcirculatory assessment showed no benefit when increasing MAP from < 60 mmHg to 60–90 mmHg in CS patients on VA-ECMO support [98]. Likewise, combined IABP and VA-ECMO support did not show any benefit on microcirculation parameters [99]. A French study found that when macrocirculation has already been restored in patients with VA-ECMO-supported refractory CS, increasing dobutamine (above 5 μg/kg/min) or ECMO flow did not further improve microcirculation [100] even if it did improve macrocirculation.

Finally, in a very small study, assessing sublingual microcirculation in six patients with pre-shock due to ST-element elevation myocardial infarction (STEMI) treated with primary PCI, Impella LP2.5 significantly improved microcirculation parameters compared with the non-support group [101]. Remarkably, restoration of the systemic microcirculation occurred within 24 h of Impella support.

## Conclusion

Cardiogenic shock is characterized by microcirculatory dysfunction. Restoration of macrocirculation parameters is the primary goal in the management of CS. However, one goal of therapy for CS should also be the restoration of microcirculatory blood flow and thus recover oxygen supply to sustain cellular function. Recent devices such as hand-held vital microscopy, and also “easy to use, easy to learn” priceless perfusion parameters (such as capillary refill time and mottling) have been established as reliable tools for assessing microcirculation alteration during CS. Although the relationship between the persistence of microcirculation abnormalities and prognosis seems established in CS, further studies are needed to better define in which patients, in which timing, under which monitoring, patient’s microcirculation disturbances should specifically be treated in cardiogenic shock.

## Data Availability

Not applicable.

## Abbreviations

AHF: Acute heart failure
AMI: Acute myocardial infarction
AMICS: Acute myocardial infarction complicated by cardiogenic shock
BP: Blood pressure
CI: Cardiac index
CO: Cardiac output
CRT: Capillary refill time
CS: Cardiogenic shock
GMP: Guanosine monophosphate
IABP: Intra-aortic balloon pump
ICU: Intensive care unit
LVEF: Left ventricular ejection fraction
MFI: Microcirculatory flow index
NIRS: Near-infrared spectroscopy
PPV: Proportion of perfused vessel
PVD: Perfused vessel density
SCAI: Society for Cardiovascular Angiography and Interventions
SBP: Systolic blood pressure
TVD: Total vessel density
VA-ECMO: Venoarterial extracorporeal membrane oxygenation
